# Supplementary material for: Are carbon emissions trading and green financial instruments synergistic? -Comprehensive quantitative research based on content analysis
Source: PLoS One. 2024 Mar 7;19(3):e0298601. doi: 10.1371/journal.pone.0298601 (PMC10919601; doi:10.1371/journal.pone.0298601)
Supplement: S1 Data — (ZIP) [file pone.0298601.s001.zip › Data/地级市Z_F工作报告.zip!/║╙▒▒/╟╪╗╩╡║╩╨/└┤╫╘╣1⁄2╓┌║┼í╛┬φ┐╦╩2╛▌═°í┐_2019.docx]

各位代表：

现在，我代表市人民政府向大会作政府工作报告，请予审议，并请各位市政协委员和列席人员提出意见。

一、2018年工作回顾

刚刚过去的一年，是全面贯彻落实党的十九大精神开局之年。我们以习近平新时代中国特色社会主义思想为指导，认真贯彻党中央、国务院和省委、省政府决策部署，在市委的坚强领导下，坚持稳中求进工作总基调，践行新发展理念，把握高质量发展要求，深化供给侧结构性改革，按照省委“三六八九”工作思路，深入实施生态立市、产业强市、开放兴市、文明铸市发展战略，经济运行呈现稳中有进、进中提质的良好态势，沿海强市、美丽港城和国际化城市建设迈出坚实步伐，较好完成了市十四届人大三次会议确定的目标任务。

——经济增速稳步加快。初步核算，全年实现地区生产总值1635.56亿元，比上年增长7.3%，增速自2016年末以来均保持在7%以上。规模以上工业增加值增长8.8%，居全省第2位；工业投资增长12.2%，工业技改投资增长14%；社会消费品零售总额增长8.8%。

——产业结构不断优化。三次产业结构调整为12.4∶33.2∶54.4 ；服务业对经济增长贡献率为60.2%，拉动经济增长4.4个百分点；装备制造业增加值增长14.2%，对规模以上工业增加值增长的贡献率达到53.9%；高新技术产业增加值增长14.6%。农业产业化经营率连续十年保持全省领先。

——质量效益大幅提高。全部财政收入、一般公共预算收入均增长12.6%，税收占一般公共预算收入的比重达到70.1%。规模以上工业企业利润总额130.11亿元，增长31.2%，总量和增速分别居全省第7位和第3位。

——生态治理成效显著。PM2.5平均浓度下降13.6%。饮用水水源地和近岸海域功能区水质达标率均达100%，北戴河海水浴场水质一类标准比例近100%。高质量荣膺国家森林城市称号，高水平承办省第二届园博会。

——人民生活持续改善。民生支出占一般公共预算支出的87.8%，增长11.9%。居民消费价格上涨2.2%。城镇和农村居民人均可支配收入分别增长7.9%和9.2%，均高于经济增速。青龙实现高质量脱贫摘帽，旅游旺季工作实现“好上加好”，我市在“中国地级市民生发展100强”中排名跃升至第25位。

一年来，我们突出抓重点、补短板，三大攻坚战开局良好。强化目标导向和问题导向，周密部署、精准施策。防范化解重大风险取得重要进展。全面排查金融、债务、房地产等重点领域风险隐患，加强监测预警和早期干预处置，严厉打击违法违规金融活动，依法依规处置了一批风险点。金融机构本外币存、贷款余额分别比年初增长13%和13.2%。加强政府债务管控，标本兼治控制新增债务、主动作为化解存量债务，争取新增债额度42.6亿元、置换债券资金26亿元。精准脱贫攻坚战取得决定性胜利。市财政每年拿出5000万元资金支持青龙脱贫。下大力抓好国家、省扶贫成效考核反馈意见整改。统筹产业发展、教育培训、健康医疗、社会保障等多种手段，精准实施“百企帮百村”等系列行动，33个易地扶贫搬迁安置小区建成入住，108支驻村工作队接续奋斗，青龙贫困发生率由2016年初的10.17%下降至0.66%，青龙县政府扶贫效率进入全国十强，全市91.37%的贫困人口稳定脱贫。污染防治攻坚战取得明显成效。启动大气、水、土壤污染防治三年作战计划。实施9家重点企业超低排放改造和深度治理，淘汰燃煤锅炉1019台，整治“散乱污”企业352家，按照“宜气则气、宜电则电、洁净煤托底”原则，完成清洁取暖改造1.2万户。全年空气质量达标天数285天，比上年增加17天。深化河长制、推进湾长制，治理涉水企业408家，取缔“三无”船舶1454艘，成功消灭重点河流劣五类水体和城市黑臭水体。强力推进露天矿山综合整治，修复责任主体灭失矿山22座。完成造林42.6万亩，森林覆盖率达到54%。

一年来，我们坚决去产能、转动能，供给侧结构性改革成效明显。把提高供给体系质量作为主攻方向，出台实施系列三年行动计划，坚决去、主动调、加快转，发展的韧性活力持续增强。加大“去”的力度。压减炼铁产能250万吨、炼钢产能300万吨，提前完成“十三五”化解过剩产能任务。安丰公司、宏兴公司等钢铁企业加快转型步伐，首秦公司向转型为“首钢赛车谷”迈进。加快“转”的速度。实施省市重点建设项目105项，固定资产投资增长4.9%。中信戴卡轻量化车轮绿色智能生产线投入运营，鹏鼎科技高阶HDI印制电路板扩产项目建设完成，抚宁120万千瓦抽水蓄能电站、哈电通用电气9H级重型燃机生产线等项目正式开工。北戴河新区生命科学园、国际健康中心等46个重点项目顺利推进。康复辅具产业园正式启动，我市被确定为国家康复辅助器具产业创新大会永久会址。把握“质”的要求。巩固全国质量强市示范城市创建成果，兴龙轮毂商标被认定为中国驰名商标，秦皇岛开发区被认定为国家级绿色园区，骊骅淀粉公司被认定为国家级绿色工厂，中信戴卡六号线被评为国家级智能制造和人工智能试点示范项目。推动“兴”的步伐。深入实施乡村振兴战略，加快农业供给侧结构性改革，调减大田玉米17.65万亩，新建果品基地10万亩，省级农业产业化示范联合体达到11家。开展农村人居环境整治三年行动，改造农村危房8034户、农村厕所3.3万座，农村垃圾治理市场化率达到100%。常住人口城镇化率达到59.42%。

一年来，我们不断深化改革、扩大开放，发展动力活力有效激发。以实际行动庆祝改革开放40周年，着力提升改革开放的系统性、整体性、协调性，推进重点领域改革向纵深发展，对外开放全方位扩大。不断深化重点领域改革。积极推进“放管服”改革，实施“一章制”审批，在全省率先实现“58证合一”，市本级“最多跑一次”事项达574项，工程建设项目和企业开办审批时间压缩至110和5个工作日，全年新增市场主体2.52万个。市县两级机构改革顺利完成。财政预算绩效管理改革加快推进。国有企业“三供一业”分离移交任务基本完成，市级经营性国有企业实现集中统一监管。扎实推进“双创双服”活动，33个办理组累计领办代办项目260个，解决问题1500多个。实施简政减税降费政策100余项。成功开展全省银行业走进秦皇岛活动，与11家金融机构签订战略合作协议，投放贷款271.27亿元。预计全年民营经济增加值比上年增长7.5%以上。坚定不移走开放融通、合作共赢之路。成功举办中国康复辅助器具产业创新大会、北戴河生命健康产业发展论坛暨第二届生命科学峰会、河北省海归博士基层创新创业行暨秦皇岛国际英才创业周等系列活动，一批重大项目成功签约，全年实际利用外资11.09亿美元，增长8.5%。秦皇岛开发区跻身全国国家级开发区综合排名30强。精准融入京津冀协同发展，实施与京津合作项目153项，与北控集团、首创集团的合作进一步深入，京能热电联产项目工程过半，北京京威股份整体搬迁项目投产达效。主动融入“一带一路”建设，韩中蒙国际海铁联运集装箱班列开通运营。制定十项针对性措施，积极应对中美贸易摩擦。全年实现出口总额237.16亿元，增长10%。深入实施创新驱动发展战略。推进科技倍增计划，高新技术企业和科技型中小企业分别达到197家、2429家，星箭玻璃等17家企业入选省“专精特新”中小企业，市级以上众创空间达到35家，清华大学智能装备研究院仪器成果转化平台投入使用，院士工作站达到19家。实施知识产权“三优”培育工程，万人发明专利拥有量7.9件，居全省首位。中瑞设计港如期开园，入驻企业22家。实施顶尖人才、科技领军人才工程和港城英才计划，聘请100名专家为人才大使。

一年来，我们强力促转型、提品质，一流国际旅游城市建设迈出坚实步伐。以港口转型升级为契机，完善顶层设计，落实体制机制，旅游产业层次和城市功能品质得到全面提升。高起点加强规划引领。以推进秦皇岛港西港区转型为目标，成功承办首届河北国际城市规划设计大赛。秦皇岛市规划纲要和北戴河地区规划、秦皇岛港总体规划、西港片区控制性详细规划和城市设计等重点规划编制工作全面展开，国际旅游城市建设行动计划纲要、全域旅游发展规划编制完成，一流国际旅游城市规划体系加快构建。高站位推动港口转型。启动西港新城、邮轮港口建设，国际旅游港起步区一期工程初具规模，海誓花园、游船码头等项目投入使用，1200亩近海公园正式开放，西港工业遗产项目成功入选国家工业遗产名录。高水平推进旅游产业发展。精彩举办首届市旅发大会，总投资292亿元的13个旅游项目签约落地。佳龙山海关文化旅游度假区、悦榕庄度假酒店综合体等项目加快推进，完成心乐园、华夏颐养众享庄园等一批新业态旅游项目，拉开了秦皇岛旅游“上山、下海、进村”的大框架，秦皇山海、康养福地的旅游形象进一步提升。接待国内外游客突破6200万人次，实现旅游总收入825亿元，分别增长18.98%和25.3%。高标准建设管理城市。开展城市面貌综合提升三年攻坚行动，累计整治“双违”27164处、1639.5万平方米；102国道市区改建工程开工，北戴河机场快速路、沿海公路石河桥至冀辽界段建成通车，河北大街、和平大街铁路桥工程竣工，打通“断头路”9条。深入推进交通秩序综合整治工程，完成城市区22处交通节点、9条主干道路的优化改造，获评全省最畅通城市。改造完成老旧小区88个，棚户区改造开工9390套，城市区新改建厕所183座，困扰多年的范家店片区征拆基本完成。深入实施城市“双修”，加快栖云山生态修复，仅用10个月时间建成大型综合性园博园，入园人数逾百万人次，成为秦皇岛的标志性景区、城市新客厅和旅游休闲新高地。

一年来，我们着力解难题、惠民生，群众获得感幸福感显著增强。坚持尽力而为、量力而行，在发展中增进民生福祉，为民实事十大工程全面完成。持续提高就业和社保水平。实施失业保险援企稳岗“护航行动”，发放稳岗补贴9066.38万元，城镇新增就业5.99万人，登记失业率2.82%。社会保险覆盖面持续扩大，城乡居民低保标准、特困人员基本生活标准、企业退休人员基本养老金水平进一步提高。持续加快社会事业发展。大力压缩一般性开支，筹措资金2亿元，支持城乡义务教育一体化和基层医疗机构达标。新改扩建中小学幼儿园30所，新改造农村义务教育薄弱学校39所，累计撤并教学点112个。成立2个医疗集团、9个医共体，全市二级以上公立医院全部建立现代医院管理制度，全面落实基本药物零差率销售政策，新改建乡镇卫生院、社区卫生中心25所，配备先进诊疗设备74台。市博物馆完成主体工程，奥体中心冰球馆、综合馆建成投用，成功举办国际马拉松、全国帆船冠军赛、中式台球国际大师赛等大型赛事，在省第十五届运动会上取得历史最好成绩。持续加强社会治理能力建设。强势开展扫黑除恶专项斗争，打掉涉黑涉恶团伙30个。零容忍打击传销，摧毁4个特大传销组织、1069个传销窝点。退役军人管理服务机构实现市、县、乡、村四级全覆盖，在全省率先完成退役军人数据信息采集，退役军人优待政策全面落实。170个房地产开发遗留问题清零，10万余户群众的办证难、入住难、征拆难问题得到妥善解决。全市信访总量大幅下降。村、社区“两委”换届圆满完成，综合排名全省第一。深入开展安全生产大排查大整治攻坚行动，加强安全生产双重防控体系和基层基础建设，未发生较大以上安全生产事故。森林防火取得无省市挂账火灾历史最好成效。食品药品安全专项整治成效明显，社会保持和谐稳定。

一年来，我们切实强法治、转作风，政府自身建设得到新的加强。坚决落实全面从严治党要求，大力推进政府系统党的建设。认真做好中央、省委巡视反馈意见整改，严格落实中央八项规定及其实施细则精神，深入开展“一问责八清理”、纠正“四风”和作风纪律专项整治，干事创业精气神焕然一新。加强法治政府建设，认真学习贯彻宪法，积极推进地方立法，严格行政执法监督，全面增强政府依宪决策、依法行政能力。自觉接受人大法律监督、政协民主监督和社会监督，181件人大代表建议、348件政协提案全部办复。国防动员和双拥共建工作深入开展，民族宗教、新闻出版广电、外事侨务、人民防空、气象地震、地方志、档案、审计、统计、援疆、妇女儿童、老龄、残疾人等事业都有了新的发展和进步。

各位代表，过去一年，我市既处于转型升级阵痛期、爬坡过坎关键期，又面临外部环境风险挑战骤然增多的严峻形势，取得这样的成绩殊为不易。这是习近平新时代中国特色社会主义思想科学引领的结果，是市委总揽全局、正确领导的结果，是市人大、市政协及社会各界鼎力支持的结果，是全市人民团结一心、砥砺奋进的结果。在此，我代表市人民政府，向全市人民，向人大代表、政协委员，向各民主党派、工商联、无党派人士和人民团体，向驻秦人民解放军、武警官兵、政法干警和消防救援队伍指战员，向中省直机关驻秦单位，向关心支持秦皇岛发展的海内外朋友们，致以崇高的敬意和衷心的感谢！

安不忘危，兴不忘忧。我们清醒地认识到，当前经济运行稳中有变、变中有忧，风险和困难增多。“三期”叠加影响仍在持续，结构调整阵痛犹存，发展不平衡不充分问题依然突出；财政增收与刚性支出需求矛盾凸显，防范化解重大风险还需持续发力；创新能力不强，创新驱动尚未成为发展的主动力；区域经济实力亟待提升，各类开发区龙头带动作用发挥不够；实体经济困难较多，金融和实体经济的良性循环尚未形成；企业和群众办事难问题仍然突出，营商环境还不尽人意；环境保护任务依然艰巨，生态建设还需加大力度；民生领域仍有不少短板，优质公共服务供给不足。与此同时，政府工作的质量和效率有待进一步提升，干部队伍干事创业、担当作为的激情还没有充分激发，隐形变异的形式主义、官僚主义和消极腐败现象不同程度存在。面对这些矛盾和问题，我们将本着对党和人民高度负责的态度，强化目标导向和问题导向，认真加以解决。

二、2019年工作总体要求

今年是新中国成立70周年，是全面建成小康社会、实现第一个百年奋斗目标的关键之年。综合分析，世界面临百年未有之大变局。我国发展仍处于并将长期处于重要战略机遇期，加快经济结构优化升级、提升科技创新能力、深化改革开放、加快绿色发展、参与全球经济治理体系变革带来新机遇、赋予新内涵。秦皇岛正处于重大历史机遇最为集中，新旧动能转换最为紧要的时期，“四市战略”的科学性、前瞻性不断显现，经济实力的提升、产业结构的优化，使沿海强市“强”的步伐更快；绿色发展的成效、全国文明城市创建的成果，使美丽港城“美”的品质更高；特别是一流国际旅游城市新定位的确立，秦皇岛港向国际知名旅游港和现代综合贸易港的加快转型，必将助推城市颜值更靓、价值更大，“国际化”元素更多，形成强大的发展动力和独特优势。困难中潜藏着机遇，挑战中孕育着希望。只要我们紧扣重要战略机遇期的新内涵，开拓创新、攻坚克难，就一定能够在新一轮竞争中，化危为机、转危为安，开创新时代沿海强市、美丽港城和国际化城市建设新局面。

2019年政府工作的总体要求是：坚持以习近平新时代中国特色社会主义思想为指导，全面贯彻党的十九大和十九届二中、三中全会精神，深入落实习近平总书记对河北工作重要指示批示和中央经济工作会议、省委九届八次全会、省“两会”、市委十二届三次全会部署，统筹推进“五位一体”总体布局，协调推进“四个全面”战略布局，坚持稳中求进工作总基调，坚持新发展理念，坚持推动高质量发展，坚持以供给侧结构性改革为主线，坚持深化市场化改革、扩大高水平开放，深入实施生态立市、产业强市、开放兴市、文明铸市战略，加快建设现代化经济体系，继续打好三大攻坚战，深入开展“双创双服”活动，统筹推进稳增长、促改革、调结构、惠民生、防风险工作，保持经济运行在合理区间，进一步稳就业、稳金融、稳外贸、稳外资、稳投资、稳预期，提振市场信心，增强人民群众获得感、幸福感、安全感，强基固本，狠抓落实，保持经济持续健康发展和社会大局稳定，为全面建成小康社会收官打下决定性基础，以优异成绩庆祝中华人民共和国成立70周年。

2019年经济社会发展主要目标是：地区生产总值增长7%左右；一般公共预算收入增长7%左右；规模以上工业增加值增长6%；固定资产投资增长7%；社会消费品零售总额增长9.5%；进出口总额增长5%，出口总额增长5%；城镇登记失业率控制在4.5%以内；城镇和农村居民人均可支配收入分别增长7.5%和8%；居民消费价格涨幅控制在3%左右；森林覆盖率达到57%以上；万元生产总值能耗、PM2.5平均浓度下降和化学需氧量、二氧化硫、氨氮、氮氧化物减排均完成省下达的目标任务。上述目标的设定，综合考虑了国内外经济环境、我市经济发展态势和各方面支撑条件，既立足于稳中求进的总基调，又坚定落实高质量发展的总要求；既考虑了破解发展中矛盾问题的客观需要，又考虑了保障改善民生的必然要求；既着眼当前保持经济持续健康发展的需要，也兼顾长远与建设沿海强市、美丽港城和国际化城市目标相协调，是经过反复测算和研究论证作出的安排，是必要的、可行的，也是通过努力拼搏可以完成的。

实现上述目标，必须坚定不移坚持党对经济工作的领导。党中央集中统一领导是最大的政治优势和制度优势，是做好一切工作的定海神针。我们要学懂弄通做实习近平新时代中国特色社会主义思想，树牢“四个意识”，坚定“四个自信”，做到“两个维护”，坚决贯彻落实中央、省决策部署，在市委的坚强领导下，确保政令畅通、执行有力、令行禁止，推动我市经济社会始终沿着正确方向和道路向前发展。必须坚定不移扭住发展第一要务。发展是硬道理，是解决一切问题的基础和关键。我们必须坚持以经济建设为中心不动摇，准确把握稳中求进总基调，统筹推进“六稳”工作，落实新发展理念，坚决打好三大攻坚战，积极融入河北“三件大事”，扎实推进系列三年行动计划，加快建设一流国际旅游城市，以基础之“稳”保障发展之“进”，以秦皇岛之“进”服务全国、全省大局。必须坚定不移推进改革开放。深化改革、扩大开放，是化危为机、转危为安的关键一招。我们要拿出勇为人先的胆略深化市场化改革，贯彻供给侧结构性改革“巩固、增强、提升、畅通”八字方针，全面提高供给体系质量，纵深推进“放管服”改革，全面优化营商环境、激发市场活力；要拿出舍我其谁的气魄加快高水平全方位开放，坚持商品要素开放与规则制度开放并举，引进来与走出去并重，用好国际国内“两个市场”“两种资源”，在更大范围、更广领域、更高层次参与全球资源配置和市场竞争。必须坚定不移实施创新驱动发展战略。创新是第一动力，创新决胜未来。我们要把发展的基点放在创新上，深化科技体制改革，创新科技评价制度，加快推进以科技创新为核心的全面创新；坚持问题导向、需求牵引，做强创新主体，汇聚创新资源，提升创新能力，在成果转化上见实效，在开放创新上拓空间；强化人才是第一资源的理念，完善人才培育引进体系，推进大众创业万众创新，使秦皇岛成为创新的蓝海、创业的热土、创客的乐园。必须坚定不移树牢以人民为中心的发展思想。人民是历史的创造者，人民是真正的英雄。我们要把人民对美好生活的向往作为奋斗目标，坚持发展为了人民，把实现好、维护好、发展好最广大人民根本利益作为一切工作的出发点和落脚点；坚持发展依靠人民，尊重人民的主体地位和首创精神，充分激发蕴藏在人民群众中的创造伟力；坚持发展成果由人民共享，聚焦群众关切，担起主体责任，着力解决热点难点问题，努力为秦皇岛人民创造更加美好、更加幸福的生活。

三、2019年主要任务

今年改革发展稳定的任务艰巨繁重，必须把思想和行动统一到党中央国务院、省委省政府和市委的决策部署上来，坚持政治引领、稳中竞进、创新驱动、改革开放、保障民生、高质量发展，重点做好八方面工作：

（一）推动制造业高质量发展。紧紧扭住经济建设这个中心，深化供给侧结构性改革，推动产业链、技术链、创新链迈向中高端，做强做优“四大两特”产业，筑牢高质量发展的产业支撑。坚定不移化解过剩产能。巩固“三去一降一补”成果，严格执行质量、环保、能耗、安全等标准，统筹运用政策手段、市场规则和倒逼机制，关小促大、保优压劣，推动总量去产能向结构去产能转变，为高质量发展腾出更好资源环境和更多市场空间。推动传统优势产业转型发展。深入开展“千企转型”行动，推动工业企业转向创新驱动、绿色发展、现代管理。切实用好技改综合奖补等优惠政策，引导产业链优势企业实施新一轮技术改造。做强传统优势产业链，以安丰、宏兴公司为重点，加快冷轧薄板等项目建设，推动金属压延业向装备制造延伸对接；加快耀华集团高硼硅防火玻璃生产线等项目实施，推动玻璃产业向多功能复合型、智能化创新型方向拓展；支持金海粮油、正大食品、骊骅淀粉等企业实施技术改造，推进粮油食品业向精深加工延伸；以葡萄小镇、金士红酒养疗庄园、仁轩酒庄等为模板，培育“红酒+大健康+大旅游”发展模式。推动装备制造业高端发展。依托哈电重装等骨干企业，加快重型燃机制造基地等项目建设，筑强临港高端装备制造业优势；支持中铁山桥、新铁德奥等整合资源发展，争取齐二数控机床、北京巴威新型锅炉生产基地等项目落地，提高装备制造业整体水平。依托中信戴卡、威卡威等优势企业，加强产业链协同配套，力促汽车零部件产业向千亿级迈进。推动战略性新兴产业创新发展。发挥北戴河生命健康产业创新示范区优势，加快石药集团生物研发基地等高端项目实施，着力构建“医药养健游”大健康产业链；推进“一中心四平台”建设，打造康复辅具特色产业基地；借力国家海洋经济创新发展示范城市试点，拓展延伸海洋工程装备、海洋生物等产业链条。加强与中国电科、中船重工等军工科研院所深度合作，支持星箭玻璃公司、际华军民产业园等发展壮大，争创军民融合创新示范区。推动制造业与服务业融合发展。实施“千企上云”工程，推动工业互联网平台建设，推广“互联网+先进制造”新模式；发挥中瑞设计港带动作用，打造“设计+”产业链，以“大智移云”和工业设计助推“秦皇岛制造”向“秦皇岛创造”跃升。大力发展临港物流业，培育建设以冷链物流为主的粮食、水果、肉类集散基地。支持金融科技、高端研发、智慧物流等生产性服务业加快发展，促进制造企业向创意、研发、售后等产业链的两端延伸。实施制造强市、质量强市和标准化战略，推进标准、质量、品牌、信誉联动提升，努力使“秦皇岛制造”更具竞争优势、更具品牌价值、更具质量信誉。

（二）持续扩大内需潜力。抓住用好国家培育壮大国内市场契机，积极扩消费、促投资、强实体，以内需潜力的不断释放，巩固经济稳中有进的态势。以降本增效助推实体经济发展。切实把发展经济的着力点放在实体经济上，搞好第四次全国经济普查，加强宏观经济运行预测预判。落实国家更大规模减税和更明显降费政策，对小微企业和科技型初创企业实施普惠性税收减免，降低社保缴费率，降低用能和物流成本，清理和规范各种附加收费，进一步促进实体经济降成本、微观主体增活力。实施大企业培植计划、中小微企业成长工程，确保年内新增规模以上工业企业40家，省级“专精特新”中小企业达到40家。以有效投资增强发展后劲。深入开展重点项目建设质量年活动，完善调度督导、观摩考核项目推进机制，严格落实连续两月末位约谈、连续三月末位追责、年底末位一票否决等奖惩机制。实施重点建设项目100个以上，大力推进抚宁抽水蓄能电站等一批产业项目，加快推进佳龙山海关文化旅游度假区、万豪酒店等一批高端旅游项目，加速推进火车站片区改造、协鑫燃气电厂等一批提升城市承载能力项目，谋划实施一批农业农村领域补短板项目，加快形成储备一批、开工一批、竣工一批的项目建设梯次衔接新格局。以高质量供给扩大消费需求。积极培育信息消费、文化消费、体育消费等新增长点，促进智能消费、定制消费等新兴消费，支持电商创业平台建设。推进“增品种、提品质、创品牌”行动，推动万达商场、茂业商厦开业运营，积极探索设立免税店和港货销售店，全面提高消费服务供给质量。发挥会议会展对于消费的带动作用，办好第四届中国康养产业发展论坛、第二届中国康复辅助器具产业创新大会、京津冀国际银发产业峰会，积极争取全国海上无人系统公开挑战赛等重大活动赛事落户我市。强化房地产市场监管和预期引导，促进房地产市场平稳健康发展。开展重点行业和领域放心消费创建活动，让老百姓吃得放心、穿得称心、用得舒心。以区域经济突破推动整体实力跃升。聚焦城区经济，突出服务城市经济发展和总体功能，以西港片区、金梦海湾片区、火车站片区为重点，抓好城市中心商务区建设和楼宇经济、总部经济发展，积极引入金融、科技、结算中心等高端企业，提高城市经济密度。聚焦县域经济，因地制宜培育主导产业，壮大龙头企业，打造特色鲜明、体系完善的立县产业集群，实现在全省的增比进位。聚焦开发区经济，促进开发区回归经济建设主战场，打响开发区跨越发展攻坚战役，实质性运作开发区集团，做强以秦皇岛开发区、北戴河新区为骨干，各县区省级以上开发区为支撑的项目建设承载平台。探索试行新批工业用地“标准地”制度，建立市级领导和各部门包联开发区机制，全面推行与战略投资者合作开发模式，以区域经济突破带动全市经济“稳”得更扎实、“进”得更强势。

（三）扎实推进一流国际旅游城市建设。坚持世界眼光、国际标准，强化规划设计引领、港口转型带动，全力打造京津冀城市群特色节点城市。以高水平规划引领发展格局。突出前瞻性、整体性、协调性的要求，聘请国内外优秀专业团队，高标准规划设计秦皇岛全域，科学划定城镇开发边界，着力构建“一城一港多组团”的空间格局。加快编制国土空间总体规划，修改完善城乡规划体系，高质量编制完成秦皇岛港总体规划、北戴河地区规划、西港片区控详规和城市设计。以港口转型拓展开放领域。坚持以城定港、港城融合，聚焦高端旅游、跨境大宗贸易、战略新兴服务三大现代临港功能，全力推进港口转型升级，让港口转型成为推动城市发展由浅滩走向深海、由国内迈向国际的战略引擎。高端定位西港片区，支持河港集团加快西港新城区综合开发，做强国际旅游港起步区一期，改造提升南山片区，启动建设河北航运中心，大力发展邮轮游艇、保税购物等产业，拓展游艇帆船全产业链，打造国际知名旅游港。全力支持东港区产业转型，重点发展集装箱综合中转业务、跨境大宗商品出口保税综合业务等临港产业，积极争取汽车、冰鲜水产品等大宗货物进口资质，构建以港口为中心，海陆空一体化的综合交通体系，建设现代综合贸易港。以全域旅游彰显城市特色。围绕打造国家全域旅游示范区，以“五个延伸”为路径，加快悦榕庄度假酒店综合体、阿那亚文创小镇、戴河水镇、祖山五色天阶等项目建设，努力开辟休闲渔业、邮轮游艇等海洋休闲旅游目的地，推动旅游产业由休闲观光向康养度假转型。加快“旅游+”跨界融合发展，依托百年港口等资源优势，深挖工业、港口文化内涵，支持首秦公司加快向“首钢赛车谷”转型，大力发展工业旅游；积极盘活山海关古城资产资源，引进新业态，让古城活起来、火起来；扎实开展走遍秦皇岛长城活动，保护、挖掘、活化长城文化遗产，争创长城国家文化公园，让更多人走近长城、了解长城。提升秦皇岛国际马拉松、山海关古城年博会、北戴河春节灯会等旅游淡季文体活动知名度。紧抓冬奥会筹办契机，推动秦皇岛冰雪嘉年华等活动，发展冰雪旅游等相关产业，提高群众性冰雪运动水平。支持青龙高水平举办第二届市旅发大会。深化旅游标准化试点工作，推动智慧旅游“一网三平台一中心”建设，提升北戴河游客服务中心、京津冀游客集散中心服务功能，全面提高“吃住行游购娱”服务质量，打造优质、便捷、舒心的休闲旅游环境。以精细化管理提升城市品质。加快推进102国道市区段改建、京秦高速二通道、海滨路东西延伸和北戴河区交通疏解路工程，打通惠泽街、南岭东路等“断头路”，启动火车站北站房建设。加快北戴河机场二期扩建、秦唐高速公路秦皇岛段、京秦高速北戴河新区支线等工程前期工作，谋划打开抚宁联山向海通道。推进交通秩序综合整治工程和城市道路交通文明畅通提升行动计划，创造安全、畅通、文明的道路交通环境。建设生活垃圾应急填埋场，开展市政老旧管网改造三年行动，推进抚宁市政配套与主城区互联互通。加快海阳片区、西部片区改造开发进程，全面提升城市承载能力。深化城市面貌综合提升三年攻坚行动，按照旅游旺季前、国庆节前两个时间节点，突出抓好海阳路、红旗路、和平大街、迎宾路等重点街区改造，打造一批展示城市形象的文化创意街区。巩固全国文明城市创建成果，强力推进国家卫生城市和国家食品安全示范城市创建，突出抓好市场上档达标、市容卫生提升、食品和饮水安全等重点工程，全面提升城市品质和城市价值。

（四）全面深化改革开放创新。把握高质量发展要求，坚持市场化改革方向，扩大高水平开放，当好河北沿海经济带改革开放排头兵。深入推进重点领域改革。深化金融领域改革，扎实开展金融领域风险排查化解，依法严厉打击非法集资行为，妥善处置省融投担保风险，坚决守住不发生系统性区域性金融风险底线；积极化解债务存量，坚决遏制隐性债务增量，争取新增政府债券资金，有效化解政府债务风险。深化财税体制改革，推进财政事权和支出责任划分改革。深化城市管理综合行政执法改革，进一步理清市区两级和市直相关部门之间的职责权限。深化国有企业改革，加强国有资产监管，加快市属国有企业混合所有制改革。深化生态环境保护综合行政执法改革，整合组建生态环境保护综合执法队伍。毫不动摇支持民营经济发展。认真贯彻习近平总书记在民营企业座谈会上的重要讲话精神，坚决落实支持民营经济发展的各项政策措施。在市场准入、经营许可、招投标等方面一视同仁、公平竞争，坚决打破“卷帘门”“玻璃门”“旋转门”。鼓励金融机构加大对民营经济发展扶持力度，用实中小微企业贷款风险补偿和“政银保”政策，降低企业贷款门槛；用好应急转贷资金，有效提高企业资金过桥周转效率；在全省首推“上市贷”金融政策，鼓励银行机构为我市上市挂牌企业提供纯信用贷款。构建亲清政商关系，依法保护民营企业家人身和财产安全。扩大全方位开放。坚持引进优质资源与提升本地资源统筹推进，以商招商、专业化招商、小团组拜访与产业链招商等方式多管齐下，主动出击、定向攻关、精准招商，促成一批好项目、大项目尽快签约落地。深入开展招商引资集中攻坚和竞赛活动，建立招商引资项目预审评估机制，每月通报、每季调度、年底考核，切实掀起新一轮招商引资热潮，年内实际利用外资增长8%。深度融入京津冀协同发展，突出秦皇岛开发区、北戴河新区、秦皇岛港西港区等重点平台作用，深化与北京西城区等全面合作，承接医疗、教育、文体、金融等非首都功能转移，推进京秦创新共同体建设和公共服务共建共享。积极参与支持雄安新区开发建设，为千年大计、国家大事贡献秦皇岛力量。关注中日韩自由贸易区、中欧投资协定谈判进程，及早做好对接融入工作，年内在欧洲设立联络办事机构。推动出口加工区升级为综合保税区，加快临港物流园区等开放平台建设。实施市场多元化战略，积极联通“一带一路”中欧班列北线，推动更多技术品牌优质企业到境外投资建厂。打造市场化法治化营商环境。引进国际营商环境评价体系，深化“放管服”改革，在实现项目审批和企业开办时间“双减半”基础上，按试点标准进一步压缩审批时间，加快“互联网+政务服务”应用体系建设，全面优化政务服务流程。深化“双创双服”活动，落实“双随机、一公开”，健全企业家参与涉企政策制定机制，落细领导包联企业制度，当好企业“服务员”。严厉打击假冒伪劣等违法行为，严肃查处损害营商环境现象，树立办事便捷、服务周到、安定有序、重诺守信的秦皇岛形象。加快创新驱动步伐。落实科技创新三年行动计划，深化与省科技厅会商机制，实施县区创新跃升计划，统筹企业创新、区域创新、协同创新，建立以企业为主体、市场为导向、产学研深度融合的技术创新体系。全面实施科技型企业成长、科技服务深化等九大工程，创新科研经费使用和管理方式，加大顶尖人才、科技领军人才等引进培育力度，加快燕山大学田永君院士高压科学研究中心重点实验室建设，年内新增各类研发平台30家、高新技术企业40家、科技型中小企业400家，规模以上工业企业建立研发机构的比例达到31.9%。加大知识产权保护力度，万人发明专利拥有量达到9件以上。努力使改革开放与创新驱动相伴相生、相得益彰，为秦皇岛发展增添不竭动力、更强活力。

（五）全力推进脱贫攻坚和乡村振兴。全面落实“五个振兴”要求，巩固农业农村发展优先态势和向好形势，扎实推进农业农村现代化。巩固脱贫攻坚成果。抓重点、补短板、强弱项，围绕“两不愁、三保障”目标，抓牢建档立卡贫困户、边缘户等重点群体，深入推进产业、就业、科技、教育、社会保障等扶贫，确保年内现行标准下农村贫困人口全部脱贫。开展扶贫扶志行动，增强贫困群众内生动力和自我发展能力。深化扶贫领域腐败和作风问题专项整治。建立完善精准防贫长效机制，加大对非建档立卡低收入户扶持保障力度，确保全面小康路上不让一人掉队。构建现代农业体系。落实粮食安全责任制，增强粮食可持续生产能力。深入实施农业供给侧结构性改革，扎实推进绿色优质农产品、农业领军品牌、现代农业精品园区、创新型农业企业“四个100工程”，培育发展休闲农业等新业态，每个县区打造1—2个产业融合发展新平台，促进农村一二三产业融合发展。发展质量农业，推行标准化生产，完善农产品质量安全追溯体系，继续调减以玉米为代表的大田作物。发展绿色农业，化肥、农药施用分别降低5%和3%，畜禽废弃物资源化利用率达到75%以上。发展品牌农业，实施公共、企业、产品“三位一体”品牌战略，年内超亿元农业产业化龙头企业达到35家以上，创响一批“秦”字号农产品品牌，全市有机、绿色、无公害认证产品达到310个以上，打造京津绿色鲜活农产品直供基地。发展科技农业，建设农业高新技术产业示范区，农业产业化经营率达到71%以上。推进强村富民行动。高质量完成粮食生产功能区划定和高标准农田建设任务，做好“大棚房”问题清理整治，坚决遏制农地非农化。扎实推进农村综合改革，巩固利用农村集体资产清产核资成果，抓好农村集体产权制度改革试点。推进农村新型社区、现代农业园区、农业生态功能区“三区同建”，建立建强一批乡村振兴示范区。加强农村新型经营主体建设，探索“村级连片经营”等新模式，年内新发展合作社、家庭农场各100家，培育新型职业农民800人以上。改善农村人居环境。强力推进农村人居环境整治三年行动，健全多元化投入机制，改造农村厕所10万座，巩固提升农村垃圾治理市场化率和垃圾无害化处理率。持续抓好农田水利建设，加大病险小塘坝、小水库治理力度。推进农村公路网络建设和品质提升，新改建“四好农村路”100公里。加强历史文化名镇名村、传统村落、历史建筑的抢救、发掘、保护、开发，建设一批宜居宜业宜游的美丽乡村。加快新型城镇化进程。坚持乡村振兴和新型城镇化双轮驱动，统筹城乡国土空间开发格局，建立健全城乡融合发展体制机制。大力推进县城改造提升，深化卢龙国家级新型城镇化综合试点工作，支持特色小城镇高质量发展，年内全市常住人口城镇化率突破60%。对标全面建成小康社会必须完成的硬任务，全面推进城乡共同繁荣，建设产业兴旺、生态宜居、乡风文明、治理有效、生活富裕的美好家园。

（六）不断加强污染治理和生态建设。坚决落实习近平生态文明思想，持续加大污染治理和生态修复力度，打造京津冀城市群生态标兵城市。全力以赴打赢蓝天保卫战。狠抓大气污染防治三年作战计划落实，以更高标准开展压煤、降尘、减排、节能集中攻坚，全面降低污染物排放总量。推进35蒸吨及以上锅炉超低排放改造，实现废气重点监控企业和重点污染源在线自动监控，推进港口岸电设施建设，严格落实船舶靠泊期间使用低硫燃油规定，确保PM2.5平均浓度下降到37微克/立方米以下。全力以赴打赢碧水保卫战。深化河长制，加快主要水系污水处理设施建设与改造，持续改善重点流域水质。加大对集中式饮用水源地保护力度，推进石河水库和引青管线连接工程，实现三大水源地互联互通，提升供水能力和综合利用水平。落实国家渤海综合治理攻坚战行动计划，推行湾长制，加强海洋资源保护和开发监管，全面开展重点海水浴场水质监测、赤潮应急消除等保障工作，确保核心区海洋生态环境安全。全力以赴打赢净土保卫战。深入开展国土攻坚行动，坚决清理闲置用地，解决好批而未供、供而未用问题。落实国家土壤污染防治计划，修复责任主体灭失矿山43个，实施尾矿、粉煤灰、钢渣等大宗固废处理与综合利用，严厉打击擅自倾倒、堆放、丢弃、遗撒危险废物等行为。巩固提升国家森林城市创建成果，加强沿海防护林保护建设，科学精准推进植树造林，加快建设森林公园、森林小镇、森林场区，全年造林40万亩以上。加强森林防火工作，提高森林火灾综合处置能力。实施山水林田湖草系统修复工程，加强重点生态区域及各自然保护地的建设和管理。开展第二次全国污染源普查，抓好中央、省环保督察“回头看”反馈意见整改落实，加大环境执法力度，加强乡镇环保网格化管理，完善全民共治、源头防治的环境治理体系，加快建设天蓝地绿水秀的美丽秦皇岛。

（七）更好保障和改善民生。坚持把人民放在心中最高的位置，持续加大民生投入，用心用力用情办好民生实事，让改革发展成果更多更公平惠及全市人民。就业是民生之本。我们要坚持把就业这个最大的民生抓紧抓好，实施就业优先政策，做好高校毕业生、退役军人、农民工、残疾人、下岗转岗职工等重点群体就业工作，年内城镇新增就业5.3万人以上。推进创业带动就业，用市场的力量创造更多就业岗位，让人们为幸福而奋斗，在奋斗中谋幸福。教育是百年大计。我们要坚持立德树人，着力培养德智体美劳全面发展的社会主义建设者和接班人。着眼让每个孩子都能享有公平而有质量的教育，深化教育综合改革，加强校长、教师队伍建设，提升乡村教师待遇。继续安排1亿元专项资金，推进教育资源优化提升三年行动计划，大力发展公办幼儿园，支持民办普惠园建设，年内新建、改建10所公办幼儿园，新增学位2000个；主城区建成中小学3所、新开工建设中小学5所，新增学位1万个；促进普通高中优质特色发展，加快北戴河健康职业学院、市职业技能实训基地建设，年内至少建成1所国际学校；支持高等教育加快发展，推动东北大学国际学院落户我市。社保是民生所依。我们要深入推进全民参保计划，推动从制度全覆盖向人员全覆盖拓展。加强对社会保险基金、医疗保险基金的监管，确保基金安全、足额发放。提高城乡居民低保标准，完善应急救助体系，使每个群众遇急有助、遇困有帮。健康是幸福之基。我们要实施“健康秦皇岛”行动计划，深入推进医药卫生体制改革，健全“四医联动”机制；继续安排1亿元专项资金，落实基层医疗机构达标三年计划，加强医联体建设，加快形成分级诊疗体系，让群众在家门口就能享受良好医疗服务。大力发展中医药事业。做好重大疾病防控工作。推进全民健身与全民健康融合发展，举办好国际马拉松、中式台球、国际帆船帆板等国际性赛事，推广夏季海上运动、春秋陆地运动、冬季冰雪运动，优化全民健身环境，创建全国全民运动健身模范市。文化是发展之魂。我们要积极培育和践行社会主义核心价值观，深挖历史文脉，弘扬文化特质，提升长城博物馆、玻璃博物馆，支持发展民营博物馆、艺术馆，加快构建现代公共文化服务体系。加强诚信宣传教育，建立完善社会诚信体系，打造诚信秦皇岛。安居方能乐业。我们要以有效改善群众居住条件为目标，建立老旧小区改造长效机制，改造老旧小区72个，棚户区改造新开工2000套。加强无障碍设施和养老设施建设，全面规范物业管理服务。坚持公交优先发展，购置新能源公交车130辆，启动城市大容量公共交通体系建设，提升县域公共交通水平。生命至上、安全第一。我们要牢固树立安全发展理念，深化重点行业领域安全生产专项整治，坚决遏制重特大事故发生，加强应急保障体系建设，提高公共突发事件应急反应和保障能力，更加有力有效维护人民群众生命财产安全。加大食品药品监管力度，确保人民群众“舌尖上的安全”。创新社会治理，强化社会矛盾排查调处，及时有效化解矛盾。加大农民工工资清欠力度，保障农民工合法权益。始终保持扫黑除恶专项斗争高压态势，深挖彻查“关系网”和“保护伞”，决不给黑恶势力以藏身之地。继续严厉打击传销活动，让传销在秦皇岛无处遁形。

做好旅游旺季服务保障工作，坚决当好首都政治“护城河”。加强国防教育、国防动员和国防后备力量建设，做好退役军人服务保障工作，维护军人军属合法权益，让军人成为全社会尊崇的职业。支持工会、共青团、妇联、社科联、科协等人民团体发挥桥梁纽带作用，做好新闻出版、广播电视、老龄、残疾人、妇女儿童、气象、防震减灾、民族宗教、外事侨务、人民防空、邮政通信、审计、统计、地方志、档案和援疆等工作，推动各项事业取得新进步。

（八）建设人民满意的政府。民之所盼、政之所向。我们一定不忘初心、牢记使命，强化宗旨意识、忠诚履职尽责，努力建设职能科学、结构优化、廉洁高效、人民满意的政府。我们要旗帜鲜明讲政治，牢固树立“四个意识”，坚定“四个自信”，坚决维护习近平总书记党中央的核心、全党的核心地位，坚决维护党中央权威和集中统一领导，严守政治纪律和政治规矩，永葆政治本色。我们要认真落实全面从严治党战略部署，深入推进政府系统党的政治建设、思想建设、组织建设、作风建设、纪律建设，把制度建设贯穿其中，层层夯实全面从严治党责任，营造风清气正的良好政治生态。我们要践行法治理念，严格遵守宪法法律，坚持科学、民主立法，依法行政、依法办事，将政府活动全面纳入法治轨道，自觉接受人大法律监督、工作监督和政协民主监督，自觉接受人民群众监督和社会舆论监督，让权力在阳光下运行。我们要不断改进作风，严格贯彻中央八项规定及其实施细则精神，驰而不息纠正“四风”，彻底摒弃“休闲文化”心态，巩固深化“两式两化”抓落实机制和“五到四从四多”工作法，让“深实严细久”成为行为习惯、行动自觉。我们要保持清廉本色，深入推进党风廉政建设和反腐败斗争，紧盯脱贫攻坚、征地拆迁、工程建设等重点领域，做好中央、省委巡视反馈意见整改落实，持之以恒正风肃纪，树立忠诚干净担当的政府形象。

各位代表！潮涌海天阔，扬帆正当时。让我们更加紧密地团结在以习近平同志为核心的党中央周围，在省委省政府和市委的坚强领导下，上下同心、攻坚克难、拼搏进取，奋力谱写新时代全面建设沿海强市、美丽港城和国际化城市新篇章，以优异成绩向中华人民共和国成立70周年献礼！
